# Editorial: Immunological precision therapeutics: integrating multi-omics technologies and comprehensive approaches for personalized immune intervention

**DOI:** 10.3389/fimmu.2025.1581238

**Published:** 2025-03-06

**Authors:** Mingyang Xue, Wenyi Jin

**Affiliations:** ^1^ School of Medicine, Kunming University of Science and Technology, Kunming, China; ^2^ Department of Biomedical Sciences, City University of Hong Kong, Hong Kong, Hong Kong SAR, China; ^3^ Department of Orthopaedics, Renmin Hospital of Wuhan University, Wuhan University, Wuhan, China

**Keywords:** immunotherapy, precision medicine, multi-omics analysis, immune signatures, personalized therapy

The integration of multi-omics technologies in immunology heralds a transformative era in the development of personalized therapeutic strategies. As immunotherapy continues to emerge as a cornerstone of precision medicine, this Research Topic brings together a rich collection of studies that explore how comprehensive multi-omics approaches—ranging from genomics and transcriptomics to proteomics, metabolomics, and single-cell profiling—can be harnessed to optimize immune interventions tailored to individual patients.

One of the key themes across the contributions is the application of single-cell sequencing to unravel the complexities of the immune landscape (Yu et al.). For example, the study on gliomas highlights how single-cell RNA sequencing (scRNA-seq) data can be leveraged to identify potential biomarkers like DAPK1, which may serve as a prognostic marker for glioma progression and therapeutic efficacy. Such insights are crucial, as gliomas remain one of the most difficult malignancies to treat due to their aggressive nature and the barriers posed by the blood-brain barrier (BBB). By utilizing multi-omics data, including scRNA-seq, the authors have demonstrated how specific immune subpopulations, such as DAPK1-expressing cells, could be used to predict patient outcomes, offering a new approach for precision therapy in glioma.

Similarly, the study on cancer stemness in ovarian cancer illustrates how multi-omics data can reveal the role of cancer stem cells (CSCs) in mediating resistance to immune checkpoint inhibitors (ICIs) (Liu et al.). Through the integration of RNA sequencing and CRISPR-based screens, the authors have identified critical genes involved in regulating cancer stemness, such as SNRPE, which negatively affects ICI response. This approach exemplifies the power of combining machine learning and genomics to predict immune responses and guide treatment decisions in cancer immunotherapy (Sun et al.).

A consistent thread running through many of the contributions is the focus on immune microenvironment characterization. (Li et al.). The work on cuproptosis-related genes in ovarian cancer investigates how the deregulation of copper-dependent cell death pathways impacts the immune landscape, with the development of a robust risk score model for predicting prognosis and immunotherapy response (Xiaorong et al.). This study adds to the growing body of literature emphasizing the importance of not only tumor-intrinsic factors but also the immune environment in determining the success of immunotherapy. Similarly, the research on thyroid carcinoma highlights the role of the epithelial-mesenchymal transition (EMT) and immune cell infiltration in cancer progression, demonstrating how these pathways can be used to refine prognostic models (Wu et al.).

The studies on lung cancer immunotherapy (Yan et al.) and the gastric cancer microbiome (Qian et al.) offer further proof of the power of multi-omics in uncovering complex disease mechanisms. In the case of lung cancer, combining genomic, transcriptomic, and proteomic data provides insights into immune-related pathways, paving the way for more personalized and effective treatment options (Li et al.). The study on the gastric microbiome goes a step further by integrating microbiota data with immune-activated transcripts, suggesting that specific bacterial species may influence immune response and tumor progression, thus offering potential targets for therapeutic modulation (Qian et al.).

Across all studies, the integration of multi-omics data with machine learning algorithms is repeatedly showcased as a tool for predicting therapeutic efficacy and patient outcomes (Sun et al.). Whether through the development of prognostic risk scores or by uncovering previously unrecognized molecular interactions, machine learning serves as a bridge between vast amounts of complex biological data and actionable insights for personalized medicine.

One of the most compelling aspects of this Research Topic is its emphasis on synergistic approaches (Amiri et al). The review on CAR-T cell therapy and CRISPR/Cas9 exemplifies how combining cutting-edge gene-editing technologies with immunotherapy can enhance treatment specificity and efficacy (Amiri et al). As CAR-T cells are increasingly used in the treatment of hematologic malignancies, the integration of CRISPR/Cas9 holds the promise of overcoming some of the limitations, such as the tumor’s ability to evade immune detection. By boosting CAR-T cell persistence and engineering them to overcome immune suppression, CRISPR-edited CAR-T therapies could expand the applicability of immunotherapy to solid tumors, offering new hope for patients with refractory cancers.

Together, these articles underscore the potential of personalized immune interventions (Wen et al.) and demonstrate the vast promise of multi-omics technologies in immunology (Sennikov et al.). They not only contribute to a deeper understanding of immune responses but also provide critical insights that will shape the future of immune-related disease treatments (Zhang et al.). As this Research Topic shows, the ability to integrate diverse types of data, from single-cell sequencing to machine learning models, allows us to build a more comprehensive and nuanced view of the immune system, its dysregulation in disease, and how best to tailor therapies to individual patients.

In conclusion, this Research Topic contributes to the growing momentum towards precision immunotherapy (Chen et al.). It reinforces the need for interdisciplinary approaches that combine the power of genomics, transcriptomics, proteomics, metabolomics, and computational techniques to drive the development of highly personalized therapies. As we move forward, these strategies will not only enhance the efficacy of existing therapies but also open the door to entirely new modalities of immune modulation that could transform the treatment of cancers, autoimmune diseases, and beyond.

